# *In vivo* measurements reveal a single 5′-intron is sufficient to increase protein expression level in *Caenorhabditis elegans*

**DOI:** 10.1038/s41598-019-45517-0

**Published:** 2019-06-24

**Authors:** Matthew M. Crane, Bryan Sands, Christian Battaglia, Brock Johnson, Soo Yun, Matt Kaeberlein, Roger Brent, Alex Mendenhall

**Affiliations:** 1 University of Washington, School of Medicine, Department of Pathology, Seattle, WA USA; 2 Fred Hutchinson Cancer Research Center, Division of Basic Science, Seattle, WA USA

**Keywords:** Gene expression, Gene regulation, Multicellular systems

## Abstract

Introns can increase gene expression levels using a variety of mechanisms collectively referred to as Intron Mediated Enhancement (IME). IME has been measured in cell culture and plant models by quantifying expression of intronless and intron-bearing reporter genes *in vitro*. We developed hardware and software to implement microfluidic chip-based gene expression quantification *in vivo*. We altered position, number and sequence of introns in reporter genes controlled by the *hsp-90* promoter. Consistent with plant and mammalian studies, we determined a single, natural or synthetic, 5′-intron is sufficient for the full IME effect conferred by three synthetic introns, while a 3′-intron is not. We found coding sequence can affect IME; the same three synthetic introns that increase mcherry protein concentration by approximately 50%, increase mEGFP by 80%. We determined IME effect size is not greatly affected by the stronger *vit-2* promoter. Our microfluidic imaging approach should facilitate screens for factors affecting IME and other intron-dependent processes.

## Introduction

Introns are noncoding DNA sequences interspersed between protein-coding DNA sequences called exons. When a gene is transcribed, introns are removed by the splicing machinery to generate a spliced messenger RNA (mRNA)^1,2^. Introns can affect gene expression in several ways (reviewed in^3^). For example, introns facilitate creation of different protein isoforms – a phenomenon known as alternative splicing^4,5^. Introns can also encode catalytic and regulatory RNAs^6–11^. And, introns may restrict genomic instability^12^. Finally, and the focus of this report, introns can increase the expression level of a gene via a number of different mechanisms. The expression level increase is referred to as intron mediated enhancement of gene expression level (IME; reviewed in^13–15^).

In general, IME is defined as the increase in expression of an intron-containing gene compared to an otherwise-identical intronless version of the same gene^16^. There are several mechanisms by which introns can increase expression levls^13–15^. In mammalian and plant systems, introns increase the rate of transcription, and this can be mediated by splicing independent functions of splicing factors such as U1 snRNA^17–19^ or by gene looping^20^. Moreover, proteins deposited onto spliced transcripts, called the exon junction complex (EJC), increase mRNA transport from the nucleus to the cytoplasm^21,22^. The EJC can also increase translation efficiency of spliced transcripts^23–25^. Experiments swapping the sequences and orientations of introns suggest the proximity of the intron to the promoter is critical for IME^26^, and not the orientation of the sequence. Rose and Gallegos created a model wherein promoter-proximal introns contain stimulatory signals controlling transcription initiation or re-initiation, in a chromatin state dependent manner, either allowing an open chromatin state, or facilitating activating histone modifications^14^.

A 5′-intron near the promoter is often sufficient for IME^27,28^. 5′-introns can increase gene expression levels by increasing recruitment of RNA polymerase II to the transcription initiation site^18,29^. Promoter-proximal introns can also affect transcription initiation via chromatin modifications^30,31^. A 5′-intron is sufficient to recruit chromatin opening marks^30^. Previous reports measured 5′-intron IME in terms of RNA abundance^27,30,32^, protein activity^27,28^ or phenotype (related directly to protein activity^33^). The contemporaneous best available technologies for these studies had technical caveats like the inability to control transgene copy number *and* locus (either of which could have affected transgene expression level^34,35^). The *in vitro* measurements of IME at the RNA and protein level were assayed via biochemical extracts^27,28^. Finally, in some cases, reporter gene expression was transient^23^ (temporally unstable due to the nature of transfection/transduction). With modern genome editing and microfluidic technologies, we are able to quantify IME in live *C. elegans* with improved technical precision.

Below we describe a microfluidic imaging system we developed to study IME *in vivo* in *C. elegans*. We designed a system that is higher throughput than confocal microscopy but still image based (unlike worm-flow)^34^, and compatible with fluorescent stereoscopes found in most *C. elegans* labs. We show the effects of different sequences and positions of natural and synthetic introns on the expression level of mCherry, controlled by the *hsp-90* promoter. We also show the effects of a different promoter (*vit-2*) and a different coding sequence (mEGFP) on IME. We compare the size distribution of introns using modern drafts of the *C. elegans* and human genomes. Understanding details of IME is important for basic biology^36^, biotechnology^37,38^, and human diseases. There are now several diseases in which mutations in introns affect gene regulation^39,40^. Utilizing *C. elegans* to understand the biology of introns *in vivo* may offer complementary, novel physiological or molecular insights.

## Results

### A cost-effective, semi-automated microfluidic device for quantifying gene expression in individual animals

**Figure 1:**
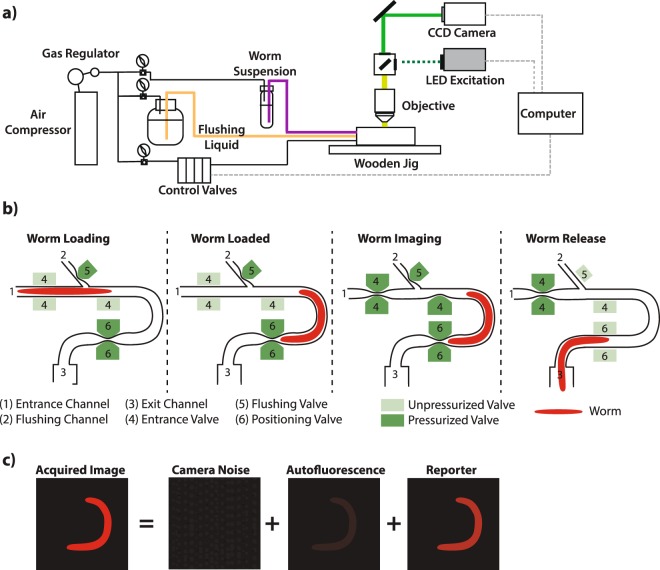
Overview of microfluidic imaging device schematics and image calibration procedure. (**a**) shows a schematic overview of the worm microfluidic measurement device. (**b**) displays a time series of cartoons showing valve openings and closings occurring during imaging of experimental groups of worms. Depth of field on our objective is approximately 55 micrometers (see Materials and Methods), and our imaging chamber is 50 micrometers deeps in z, ensuring we capture all the signal form each animal. (**c**) shows the image correction protocol we used to determine average voxel intensity, quantifying expression level as a function of concentration (not total signal); see Supplementary Fig. 1 for an image of a fluorescent worm in the imaging chamber.

We modified a previous microfluidic chip design^41,42^ to develop an instrument to quantify gene expression in whole animals while acquiring an image of each animal. In this system, air pressure moves the worms from a pressurized 1.5 mL tube into the PDMS chip where they are subsequently imaged (Fig. 1). On the chip, animals are imaged in a “U” shaped orientation, and then flow out of the imaging chamber into an exit tube. The design can accommodate manual sorting of animals with complex phenotypes by diverting the exit tube onto solid or liquid worm growth media. Figure 1a shows an overview of the setup. Figure 1b details the process of entry and exit into the imaging chamber. Figure 1c shows how we quantify signal from animals. We can typically image about 100 worms in twenty minutes. This instrument is not as fast as the Copas Biosort, but it captures an image of every worm. For our initial experiments with *hsp-90* reporters, we used exposure and excitation near the bottom of the linear dynamic range (Supplementary Fig. 1) to ensure the same settings could be used with stronger promoters/signals. We validated the dynamic range of the system using dilutions of lyophilized mCherry; the dynamic range covers a more than 50 fold range of protein concentration (Supplementary Fig. 1). We validated the microfluidic system’s results using a COPAS Biosort (worms measured in flow) and quantitative, cell-resolution confocal microscopy^34^ (see below and Supplementary Fig. 1).

### A set of *C. elegans* expressing fluorescent proteins with different intron configurations

**Figure 2:**
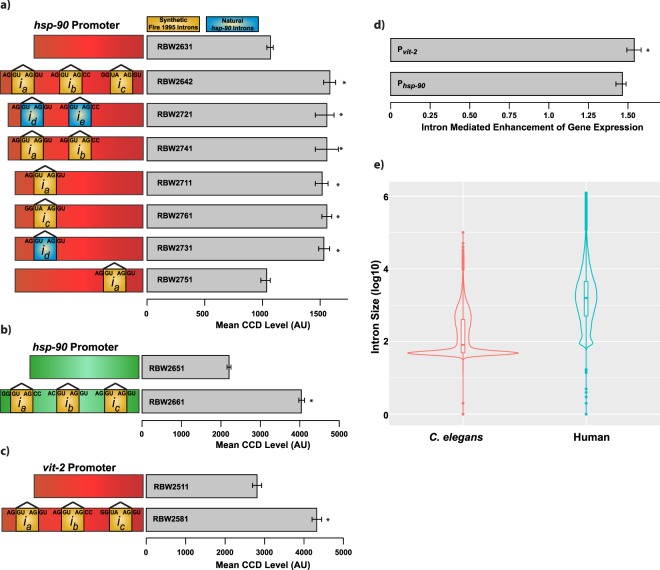
Characterization of intron sequence and location on IME from a set of transgenic *C. elegans* strains expressing fluorescent reporter proteins with and without introns from a single autosomal locus in the genome. Error bars show 95% confidence intervals. Differences between strains are therefore at a 5% significance level when the error bars do not overlap. (*) indicates strains that have a statistically significant increase relative to the no-intron containing control. Additional statistical details are provided in materials and methods and Supplementary Information. (**a**) Strains using the *hsp-90* promoter driving mCherry coding sequences that contain variable numbers and locations of introns. The exons are shown in red, and introns are shown in yellow or blue, and labeled *i*_*a-e*_. Introns *i*_*a-c*_ are the canonical synthetic introns, and *i*_*d-e*_ are the first two introns naturally found in *C. elegans’ hsp-90*. Regardless of intron sequence, or number, all strains received a ~50% boost in gene expression as long as a 5′-intron was present. A single 3′-intron was insufficient to boost expression relative to a transgene with no introns. (**b**) The protein coding sequence affects the magnitude of IME. Using strains expressing EGFP and containing either no introns, or three synthetic introns, an ~80% boost was quantified. (**c**) IME is not constrained by free translation capacity, as mCherry expressed by one of the strongest promoters also received ~50% expression boost from three synthetic introns. (**d**) There is a slight but significant increase of IME effect size when mCherry expression is controlled by the much stronger *vit-2* promoter (53.55% vs. 45.88%). (**e**) Analysis of the intron lengths in both *C. elegans* and humans.

We designed and constructed a set of eleven different *C. elegans* strains with single copy reporter genes at a Chromosome II locus, designated by a transposon insertion, ttTi5605^35,43–45^. We varied the promoter (*hsp-90* or *vit-2*), and/or number, position or sequence of introns (synthetic or natural); we used the *unc-54* terminator in all constructs. The constructs integrated into the genomes of the resulting strains are shown in Fig. 2 and listed in Table 1. The sequences of all reporter gene are listed in Supplementary Information. Because autofluorescence levels are significantly lower in mCherry, relative to mEGFP, we used mCherry for the majority of the work, except when testing the effect protein coding sequence on IME.

**Table 1:** .

Strain	Promoter	Protein	Intron One	Intron Two	Intron Three
RBW2511	*vit-2*	mCherry	absent	absent	absent
RBW2581	*vit-2*	mCherry	a^a^	b^b^	c^c^
RBW2631	*hsp-90*	mCherry	absent	absent	absent
RBW2642	*hsp-90*	mCherry	a	b	c
RBW2721	*hsp-90*	mCherry	d^d^	e^e^	absent
RBW2741	*hsp-90*	mCherry	a	b	absent
RBW2711	*hsp-90*	mCherry	a	absent	absent
RBW2751	*hsp-90*	mCherry	absent	absent	a
RBW2761	*hsp-90*	mCherry	c	absent	Absent
RBW2731	*hsp-90*	mCherry	d	absent	absent
RBW2651	*hsp-90*	mEGFP	absent	absent	absent
RBW2661	*hsp-90*	mEGFP	a^a^	b^b^	c^c^

^a^a was referred to as å in the original 1995 Fire Vector Kit plasmid pPD95.02^46^.

^b^b was referred to as ß in the original 1995 Fire Vector Kit plasmid pPD95.02^46^.

^c^c was referred to as ∂ in the original 1995 Fire Vector Kit plasmid pPD95.02^46^.

^d^d is the first intron sequence that naturally occurs in *hsp-90*.

^e^e is the second intron sequence that naturally occurs in *hsp-90*.

All strains express the transgenes from the ttTi5605 locus on Chromosome II. All strains use the *unc-54* terminator sequence.

### The canonical three synthetic introns increase mCherry expression by approximately 50%

Because of the widespread usage of the three canonical synthetic introns created by Dr. Andrew Fire^46^, we first determined the magnitude of IME using these introns. We used the constitutive, ubiquitously expressed, *hsp-90* promoter to control expression of mCherry with and without these synthetic introns. We expressed these reporter genes from the same locus on chromosome II (inserted at ttTi5605^35,44,45^). By measuring average voxel mCherry signal in our microfluidic chip (after correcting for intrinsic fluorescence in wild-type animals), we found the canonical three synthetic introns increase mCherry expression level by 46% (Fig. 2a). We confirmed our findings using two additional instruments. We found the same IME effect size (Supplementary Fig. 1) at cell resolution using a confocal microscope^34^. We also saw a similar IME effect at animal resolution (Supplementary Fig. 1) using the COPAS Biosort^34^.

### Two natural introns have the same IME magnitude as three synthetic introns

The canonical introns used in *C. elegans* reporter transgenes are synthetic, so we determined if natural introns confer IME. We replaced the synthetic introns in our *P*_*hsp-90*_*::mCherry::T*_*unc-54*_ construct with the first two natural *hsp-90* introns. We placed the natural introns at the same exon-intron boundaries as the synthetic introns, maintaining the same 5′- and 3′- splice junctions (Fig. 2). The natural *hsp-90* introns increased protein expression the same as the three synthetic introns (~50%), shown in Fig. 2a.

### A single 5′-intron is sufficient for IME

Prior reports demonstrated a single 5′-intron is sufficient for some level of IME^27,28,33^. Therefore, we tested if single 5′-introns are sufficient for IME. We generated strains containing one, two or three synthetic or natural introns at different positions in the coding sequence, shown in Fig. 2a. We found that two synthetic introns produced the same IME effect as all three synthetic introns. Strains containing only a single natural or synthetic intron at the first position had the same level of IME as all three synthetic introns or both natural introns (Fig. 2a). A single intron at the 3′-site was not sufficient to increase gene expression, at least when intron “a” was used (this intron is sufficient for IME when placed 5′ (Fig. 2a)).

### Protein coding sequence affects IME magnitude

To determine if protein coding sequence affects IME, we generated strains containing the mEGFP coding sequence with and without the same three synthetic introns described above. Figure 2b shows that we observed an IME effect size of ~80%, which is significantly different from the ~50% increase in gene expression conferred by the same introns in the context of the mCherry coding sequence. To validate that IME magnitude could be dependent on the coding sequence, we measured animals on four separate days using a point scanning confocal microscope as previously described^34^. We compared IME for mEGFP and mCherry measured from the same cell (Int1DL) from ~40 animals. Using this approach, we again found coding sequence affected IME (Supplementary Fig. 1c). While there are certainly more coding sequences to test, these results suggest that coding sequence can impact IME, at least for two divergent fluorescent proteins (48% and 30% identity at the DNA and protein level respectively).

### IME magnitude is slightly affected by the *vit-2* promoter

To determine if promoter strength affects IME, we replaced the *hsp-90* promoter with *vit-2*. The *vit-2* gene encodes a yolk protein expressed in the intestine of *C. elegans*. When considering it is only expressed from the 20 intestine cells^47^, it is possibly the most highly expressed gene (in terms of concentration) in adulthood^48^, which is when we measured the animals (day one of adulthood). If IME were primarily constrained by free translational capacity, the magnitude of protein increase could depend on the transcriptional strength of the promoter. Stronger promoters would thus receive less of a boost. We constructed two strains using the *vit-2* promoter to drive mCherry with either no introns, or the three synthetic introns (Fig. 2c; Table 1). The presence of three synthetic introns increased protein expression by ~50% (Fig. 2c) showing that the distinctly regulated, and stronger *vit-2* promoter does not have a large effect on the magnitude of IME at the protein level compared to the *hsp-90* promoter (Fig. 2d). Note the absolute value of protein produced under *vit-2* promoter control was much higher. Thus, the magnitude of IME does not appear to be constrained by free translational capacity. Most promoters remain untested, and may confer larger or smaller IME effect sizes.

### Intron size distributions in *C. elegans* and *H. sapiens*

Relatively short, single 5′-introns are sufficient to increase gene expression level in plants^27,32^ and mammals^28^, and now we can add *C. elegans* to the list. The short single introns in these studies are generally representative of short introns sizes in worms and humans. Figure 2E shows the distribution of intron sizes in modern drafts of the *C. elegans* and *H sapiens* genomes. Both worms and humans have ~20,000 protein coding genes, but the human genome is about ten times bigger. Introns in *C. elegans* are generally shorter than in humans, and both *C. elegans* and *H. sapiens* have a modal cluster of short introns (Fig. 2E). However, perhaps as expected with a more compact genome, short introns compose a larger proportion of total introns in *C. elegans*.

## Discussion

The microfluidic quantification system described here allowed us to quantify whole animal reporter gene signals from live *C. elegans* at medium throughput (30–100 animals in 30 minutes). Our previous measurements of gene expression in whole animals^34,49–51^ utilized a higher throughput (300–1000 animals in similar timeframe) flow sorting device (COPAS Biosorter, Union Biometrica) that measures signal from animals as they pass by a photomultiplier tube. This instrument does not acquire images of each animal, forgoing acquisition of potentially useful image features. By contrast, our system collects an image of each animal in a fixed orientation (see Fig. S1). Our particular system’s objective lens has a large depth of field, (>50 µm) allowing collection of signal from the entire depth in z of the immobilized worm.

In this report, we found that single or multiple introns increased protein expression level by about 50% or 80%, depending on coding sequence, and these data fit within the previously reported range of IME. In Nott *et al*., 2003, a single 5′-intron was sufficient to increase gene expression by 29-fold^28^. In Bieberstein *et al*. 2012, the mouse FOS gene expressed five times more RNA when it included three natural mouse introns; they also found a single 5′-intron sufficient to cause chromatin markings associated with increased gene expression level^30^. In Rose 2002, several distinct *Arabidopsis* introns were identified, which increased expression of reporter gene RNA between 40% and 1490%^32^. Similarly, in Rose 2004, a variety of single 5′-proximal *Arabidopsis* introns were found to be sufficient for IME, ranging from 10% to 2000%^27^. Thus, the effects we measured fall between the effects that have been measured before. Most introns remain untested in *C. elegans* (see additional examples and scenarios in^52^). It is possible that future studies will identify *C. elegans* intron sequences that confer greater IME effect sizes than we found here.

Our findings corroborate results from prior *in vitro* studies showing that a single 5′-intron is sufficient for IME using phenotypes^33^, extracted RNA^27^ or extracted protein activity^27,28^ as readouts for the effect of introns on gene expression level. Our results are consistent with the idea that IME occurs via transcription, translation, or both. Our results are consistent with a transcriptional mechanism in which the splicing machinery at the 5′-intron re-recruits RNA polymerase back to the transcriptional start site. Our results are consistent with the possibility that a 5′-intron may affect transcription by opening chromatin; this may occur via recruitment of molecular machinery that makes access-permissive histone modifications to promoter-proximal histones. Our results are also consistent with the possibility that a 5′-intron can affect translation via proteins deposited onto the EJC during splicing^53,54^, which can recruit the translation machinery to EJC-marked transcripts^55^. Clearly, more work is needed to answer many lingering questions about IME^13–15,56^.

Many previous reports have shown that introns influence human diseases. These diseases fall into two broad classes: heritable diseases^39,40,57^ and cancers^58–62^. The intron-dependent processes affected are often unknown, poorly understood, or if understood, lack experimental means to determine how to suppress the intron-related pathology. For example, in a bipolar risk allele, STRs in the third intron of the calcium encoding CACNA1C gene seem to affect IME^40^. However, the mechanism by which this large, relatively 3′-intron acts to increase gene expression level remains unknown. The reagents and tools described here will allow investigators to perform genetic screens for factors affecting intron-related phenotypes. Moreover, the recent development of synthetic food medium as an alternative to the OP50 *E. coli* strain should allow execution of chemical genetic screens, using, for example, FDA approved pharmaceuticals packaged into liposomes and delivered into the intestine cells via the alimentary canal^63^. It is our hope that the screenable *in vivo* system we presented here will provide insight into intron-related disease mechanisms, and lead to therapeutic treatments to alleviate human diseases.

## Materials and Methods

### Strains and culture conditions

Here, we used wild-type N2 strain of *C. elegans*, RBW6699 and the strains we listed in Fig. 1 and Table 1: RBW2642, RBW2661, RBW2511, RBW2581, RBW2631, RBW2651, RBW2721, RBW2741, RBW2731, RBW2761, RBW2711, and RBW2751; each strain has an insert number corresponding to its strain number, meaning RBW2511 contains hutSi2511. We maintained all strains in 10 cm petri dishes on NGM seeded with OP50 *E. coli* in an incubator at 20°. Strains were maintained for at least three generations at 20° in a well-fed state before experimentation. For experiments, animals were synchronized via alkaline hypochlorite treatment followed by overnight hatchout in sterile S-basal to cause entry into the L1 diapause, described in detail in^34^. Synchronous cohorts of animals were grown to gravid adulthood (72 hours at 20° on NGM after the L1 diapause) at a density of approximately 175 animals per 10 cm NGM plate seeded with 1 mL of OP50 *E. coli* from an overnight 500 mL or 1000 mL LB growth culture.

### Generation of transgenic nematodes

To make the DNA used for the transgenes we inserted, we used a yeast homologous recombination based cloning system we described recently to generate DNA repair templates^64^. To make the reporter genes, we used 2 kb upstream of the ATG for the *hsp-90* promoter and 4 kb upstream of the ATG for the *vit-2* promoter. We used the same *unc-54* terminator in all strains^64^. For the pieces of DNA in which we varied introns, we created those with overlap extension PCR and then used those fragments in our yeast HR cloning system. These DNA repair templates were then used in microinjection-based MosSCI transgenesis to insert these reporter genes into the genome at the ttTi5605 Mos transposon locus on Chromosome II in strain RBW6699, which is an outcrossed version of EG6699 containing *unc-119(ed3)*. We injected constructs @ 50 ng/µL of repair template + coinjection and selection markers as described previously in^35,44,45^). We sequenced the reporter sequences of all DNA constructs to ensure their sequence integrity and genotyped animals to verify insertion at the ttTi5605 locus. Thus, in this way, we generated all the strains listed in Fig. 2 and Table 1, with the exception of RBW2642 and RBW2661, which were previously reported in^34^. We show the sequences of the fluorescent proteins and introns used in Supplementary Information.

### Microfluidic measurements of whole-worm gene expression levels

To prepare animals for entry into the microfluidic imaging device, we washed them from their growth plates into 1.5 mL Eppendorf tubes with filter-sterilized S-basal (to remove particulate matter) containing 0.01% tween (to prevent worms from sticking to plastic). We allowed animals to settle for 5–10 minutes in a 50 mL conical vial, and then removed the supernatant, and added fresh S-basal. We repeated this process three times to ensure that that any dust or embryos washed from the plates were removed prior to use. We then placed a “slurper” tube into the 1.5 mL Eppendorf tube. After we sealed and pressurized the Eppendorf tube and slurper lid, animals flowed into the slurper tube, and we manually loaded animals, one at a time, into the imaging chamber of the microfluidic device. We measured all strains, one worm at a time. To quantify signal from the animals in microfluidic device, we mounted the device on a wooden jig under a LED-illuminated, fluorescent Leica MFC165 stereoscope (Leica Microsystems, Wetzlar, Germany) set to 12X magnification, equipped with a 1.0x Planapo objective and a Lumenera 3–3UR camera with a 0.63X optical coupler, in a room at approximately 20°. With this setup our depth of field was approximately 55 micrometers and our xy resolution was approximately 2.7 micrometers. We focused the image on the approximate central plane of the worm body by focusing on the grinder and lumen of the pharynx, shown in Fig. 1 in^65^. Worms were physically constrained by the 65 × 50 micrometer dimensions of the imaging chamber preventing them from moving their bodies out of plane. We measured between 30 and over 100 animals per strain per experiment. We manually controlled the flow into the imaging and image acquisition in real time on the computer using Matlab and Micromanager, respectively.

### Microfluidic worm measurement device construction

Devices are constructed as previously detailed in^41,42^, using standard soft-lithography practices. Briefly, a microfluidic master mold was constructed in the University of Washington Nanofabrication facility using a transparency mask from OutputCity. The AutoCAD® design files are available at GitHub at https://github.com/nomadcrane/elegansImaging. The mold was made using SU8 2025 and following fabrication was coated with a monolayer of silane to prevent PDMS adhesion. The individual microfluidic devices were constructed using a two layer fabrication process similar to^42^ wherein a very small amount high pre-polymer to cross-linker (20:1) ratio of Sylgard 184 was poured on the microfluidic mold sufficient to cover the mold with 1–4 mm of polymer. This 20:1 ratio has a very low Young’s Modulus which allows the side valve mechanism to work. This base layer is partially cured for 45 minutes in a 70 °C oven, and then a volume of 10:1 (pre-polymer:cross-linker) Sylgard 184 is poured on top. Because the low Young’s Modulus layer is prone to damage or tearing due to the limited polymer cross-linking, this second layer provides much needed structural support for inlet hole punching and bonding. This stability layer is poured to a thickness of 15–20 mm.

Following curing of the stability layer (2 + hours in 70 °C oven), devices are cut and removed from the mold. Holes for media and valves were punched using a 1 mm diameter biopsy punch. To ensure that PDMS debris was not introduced to the flow channels as a result of the hole punching, the quality of the biopsy punch was inspected prior to use, and holes were only punched cleanly once. All holes were punched from the device side through the low Young’s Modulus flow layer. Devices were cleaned using scotch tape prior to bonding to slide glass. Bonding was performed using a Harrick PDC-001 plasma cleaner. Devices and slide glass were exposed to high energy plasma for 35 seconds, and then the device was placed on the slide glass. Bonded devices were allowed to rest for at least 1 h in the 70 °C oven prior to use.

To prevent diffusion of gas from the pressurized valves into the flow channels, valves were filled with water prior to use. This was done by connecting tubes containing DI water to the valve channels, and pressurizing the channels. The flushing media for the device used to push out the worms uses filtered M9 media containing 0.01% Triton X-100. To ensure that there was no cross-contamination between samples, the flushing media was used at moderate pressure for 5 min to rinse the device and the loading tubes. Loading and flushing pressures were optimized for each experimental run, but were typically within the range of 1–9 psi. The valve pressures were similarly optimized for individual variation in devices, but typically were used in the range of 45–60 psi.

### Valve controller box fabrication

To control both the valve pressures, and whether the valves are pressurized or released, we developed a control box composed of pressure regulators, solenoid valves, and a USB switch to provide computerized control of all on-chip valves. This system was modeled on earlier systems^41,42,66^. A complete list of parts is available on GitHub at https://github.com/nomadcrane/elegansImaging.

### Valve control software

Computerized control of both the microfluidic valve control box and the microscope camera was implemented using custom written Matlab® software. To provide generalizability, all interaction with microscope and camera was implemented using micromanager, and controlled through Matlab®. The code, is freely available on GitHub at https://github.com/nomadcrane/elegansImaging.

### Quantification of whole animal fluorescence

Image segmentation was automated to ensure consistency in quantification of expression level between different strains. Images from all strains for the same promoter, fluorescent protein combination were loaded into memory, and an appropriate segmentation threshold that minimized intraclass variance was selected using Otsu’s threshold^67^. Following subtraction of the camera noise from image, all animals containing transgenes with the same promoter/fluorescent protein combination were segmented using the same threshold. Segmented images were then opened to remove any spurious pixels. This whole animal segmentation provided consistent size measurements between strains, and had a high degree of accuracy when compared with manual segmentation of the brightfield images.

### Microscope corrections and calibrations

There are multiple sources of noise and variation that are generated during conventional epifluorescent imaging. To ensure we operated in the linear range of our camera, we quantified a mCherry dilution series we generated from lyophilized mCherry, shown in Supplementary Fig. 1. To correct for individual pixel variation in dark current noise in the CCD camera, we acquired 1,000 images using the same exposure times and binning, but without light directed to the camera. For each pixel, the median value was determined and used as the dark current pixel noise, and was subtracted from each image for all experiments. To correct for intrinsic autofluorescence, we imaged approximately 100 wild-type N2 animals using the two image acquisition settings (excitation/emission filters and camera exposure time) we used to image animals expressing mCherry or mEGFP. The whole animal auto-fluorescence was determined using both the mEGFP and mCherry parameters. There is a distribution of autofluorescence levels within the wild-type population, but for simplicity the population mean autofluorescence levels for both the mEGFP and mCherry fluorescent excitation/emission channels were calculated. These mean values were then subtracted from the transgene expressing strains to determine the magnitude of fluorescence from the fluorescent protein.

For those who care to undertake these endeavors comparing mCherry and mEGFP, we note that the intrinsic fluorescence signals emanating from secondary lysosomes and other intrinsically fluorescent (autofluorescent) bio molecules in wild-type N2 animals^34,68^ are relatively stronger when excited and imaged with mEGFP optics (and thus a greater part of the total signal measured under those optics), compared to the same images acquired using an mCherry filter set. Because of this increased background effect, to accurately determine the level of fluorescence derived from the GFP transgene, it is critical to calculate and then remove the level of autofluorescence from the imaged cells^69^ (or circumvent it by measuring an area with relatively pure reporter signal^34^).

### Cell-specific, quantitative confocal microscopy

We performed microscopy as in^34^, except that the animals were mounted in a microfluidic imaging chamber, described in^64^. Briefly, in four independent experiments we loaded animals expressing mEGFP or mCherry with or without introns, under control of the *hsp-90* promoter, into 80-lane microfluidic mounting devices. We imaged the int1DL cell in ten animals per experiment for animals expressing reporter genes with and without introns in four independent experiments. We quantified expression level of reporters using the equatorial nuclear z slice from the image stacks of each animal, described in^34^, shown in Supplementary Fig. 1.

### Measurement of worms in flow

We performed measurement of worms in flow as previously described in^34^. Briefly, we synchronized three large populations of worms as described above, and placed the starved L1s onto five 10-cm OP50-seeded NGM plates @ 175 larvae per plate. Then, after 72 hours of development, for each strain (wild-type control, RBW2631 and RBW2642), we used S-basal buffer to wash the adult animals off of the plates and into 15 mL conical vials and allowed the adults to sediment for five minutes, and then aspirated the L1-rich supernatant (the progeny of the adults) three times. We then placed these adult-enriched solutions of worms into the COPAS Biosort and measured fluorescent intensity using mCherry detection optics. We collected the data as a text file and then plotted and analyzed it using SigmaPlot 12.5 (San Jose, CA), shown in Supplementary Fig. 1.

### Bioinformatic analysis of intron sizes in protein coding genes in *H. sapiens* and *C. elegans*

To collect our data, we used GTF file for *Caenorhabditis elegans* (WBcel235 Ensembl release 94) and the human GRCh38 (Gencode v29 release GTF file). We used the Bioconductor GenomicRanges package (https://www.bioconductor.org/about/) to extract intron sizes in protein coding genes in *C. elegans* and *H. sapiens*. We plotted the log10 transformed intron size distribution for both *C. elegans* and *H. sapiens* using violin plots overlapped with boxplots, shown in Fig. 2.

### Statistical methods

We used SigmaPlot 12.5 (San Jose, CA) for all statistical analyses. We performed at least three independent experiments (different batches of animals measured on different days) for each of the strains we measured on our microfluidic device. To analyze the data, we first determined if the data was normally distributed and had equal variance using one or two-way ANOVA, and then performed appropriate statistical tests to account for multiple comparisons. We detail the major analyses used in this paper below.

The specific statistical tests and results for the microfluidic measurements are as follows: Three synthetic introns increase expression level by 45.88%; P < 0.001, Mann-Whitney Rank Sum Test. Given that we measured several different strains compared to the control, we also performed a Kruskal-Wallis One Way Analysis of Variance on Ranks followed by Dunn’s Method to compare to the intronless control and detected a significant difference P < 0.05. At least three independent experiments with at least 185 animal were performed (185–297). Natural introns significantly increase the expression level of mCherry by 47.98%; P < 0.001, Mann-Whitney Rank Sum Test. Given that we measured several different strains compared to the control, we also performed a Kruskal-Wallis One Way Analysis of Variance on Ranks followed by Dunn’s Method to compare to the intronless control and detected a significant difference P < 0.05. We performed at least three experiments with at least 179 total animals were performed (179–297 animals). With the exception of animals bearing the reporter gene with only *i*_*a*_ at the most 3′ position in the coding sequence, all other intron bearing strains expressed significantly more protein than the intronless strain; Kruskal-Wallis One Way Analysis of Variance on Ranks followed by Dunn’s Method, P < 0.05 for all comparisons. We performed at least three independent experiments with at least 100 total worms per strain (103–298 animals per group). Synthetic introns significantly increase mCherry expression level under *vit-2* control. Mann-Whitney Rank Sum Test, P < 0.001. Results are from three independent experiments with at least 130 total animals per group (134–163). The same three synthetic introns in mCherry significantly increase mEGFP expression level by 83%. P < 0.001, Two Way ANOVA followed by the Holm-Sidak method for multiple comparison procedures. Results are from three independent experiments measuring at least 200 total animals per group (215–260). Details for each of the statistical analyses performed in SigmaPlot are included in Supplementary Information.

## Supplementary information

Supplementary Infromation

## Data Availability

Expression level data is available in upon request. Strains will be sent to the CGC. Software and parts lists are available online at github.
