# Supplementary material for: In vivo measurements reveal a single 5′-intron is sufficient to increase protein expression level in Caenorhabditis elegans
Source: Sci Rep. 2019 Jun 24;9:9192. doi: 10.1038/s41598-019-45517-0 (PMC6591249; doi:10.1038/s41598-019-45517-0)
Supplement: Supplementary file 1 — Supplementary Infromation [file 41598_2019_45517_MOESM1_ESM.docx]

Supplementary Information

for

***In vivo* measurements reveal a single 5’-intron is sufficient to increase protein expression level in *Caenorhabditis elegans***

Matthew M. Crane+^1^, Bryan Sands+^1^, Christian Battaglia^1^, Brock Johnson^1^, Soo Yun^1^, Matt Kaeberlein^1^, Roger Brent^2^ & Alex Mendenhall*^1^

+ equal contribution

* To whom correspondence should be addressed: [alexworm@uw.edu](mailto:alexworm@uw.edu)

^1^ University of Washington, School of Medicine, Department of Pathology, Seattle, WA

^2^ Fred Hutchinson Cancer Research Center, Division of Basic Science, Seattle, WA

**Contents:**

Supplementary Figure 1 Page 2

DNA Sequences Page 4

Statistical Analyses Page 8

**Supplementary Figure 1**


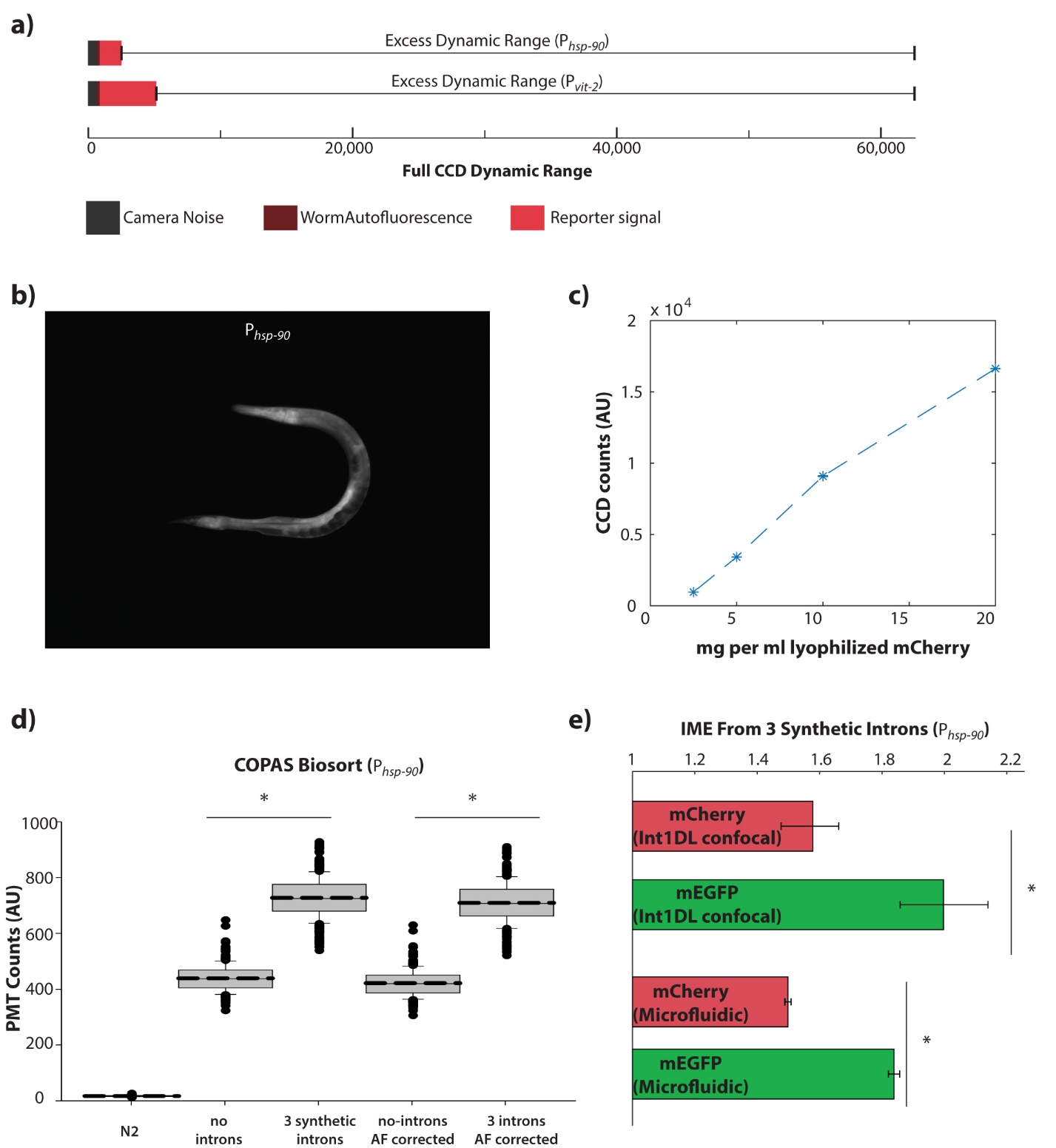


**Supplementary Figure 1. a)** A bar graph shows the contribution of different signal components in the context of the dynamic range of the camera we used. We set the camera gain and exposure for the *hsp-90* reporters to ensure excess dynamic range so the same settings could be used with the stronger *vit-2* reporters. **b)** An image of an animal expressing mCherry in the imaging chamber of the microfluidic device is shown. **c)** A line plot showing concentration of mCherry versus the CCD counts on the camera; the dynamic range covers a range of concentration exceeding 50 fold**.** To determine this, we used lyophilized mCherry to generate a series of dilutions in a black 96 well plate, and then quantified signal using the same exposure settings as the primary experiments. We measured each concentration three times, and plotted the average. We subtracted the background camera noise prior to plotting. **d)** Boxplots show fluorescent signal measured using mCherry optics for wild-type animals and animals expressing mCherry with or without introns under *hsp-90* promoter control using the COPAS Biosort. We found an IME effect of 68%, similar to the 50% IME we obtained using concentration measures with the microfluidic device (Fig. 2) or the confocal microscope (Fig. S1E). **e)** Bar graphs show the magnitude of IME conferred by the same three synthetic introns in mCherry and mEGFP, measured from whole animals with our microfluidic device, or measured from single cells with a confocal microscope. To validate the IME effects measured in the microfluidic device we used a confocal microscope to image strains with either no introns (RBW2631, RBW2651), or three synthetic introns (RBW2642, RBW2661) using the *hsp-90* promoter to drive mEGFP or mCherry. We quantified gene expression from the exact same single cell (Int1DL) measured in different animals and found similar effect sizes for IME using mCherry or mEGFP coding sequences. Error bars show 95% confidence intervals. (*) indicates a statistically significant difference at this threshold.

**DNA Sequences**

DNA sequences of reporter genes.

Coding sequence in Caps. Introns in lower case. Stop codons omitted.

mCherry no introns

ATGGTCTCAAAGGGTGAAGAAGATAACATGGCAATTATTAAAGAGTTTATGCGTTTCAAGGTGCATATGGAGGGATCTGTCAATGGGCATGAGTTTGAAATTGAAGGTGAAGGAGAAGGCCGACCATATGAGGGAACACAAACCGCAAAACTAAAGGTAACTAAAGGCGGACCATTACCATTCGCCTGGGACATCCTCTCTCCACAGTTCATGTATGGAAGTAAAGCTTATGTTAAACATCCGGCAGATATACCAGATTATTTGAAACTTTCATTCCCGGAGGGTTTTAAGTGGGAACGCGTAATGAATTTTGAAGACGGAGGAGTTGTTACAGTGACGCAAGACTCAAGCCTCCAAGATGGAGAATTTATTTATAAAGTCAAACTTCGAGGAACGAATTTCCCCTCGGATGGACCTGTTATGCAGAAGAAGACTATGGGATGGGAAGCTTCAAGTGAAAGAATGTACCCTGAAGACGGTGCTCTTAAGGGAGAGATTAAACAACGTCTTAAATTGAAAGATGGAGGACATTACGATGCTGAGGTGAAGACAACTTACAAAGCCAAAAAACCAGTTCAGCTGCCAGGAGCGTACAATGTTAATATTAAACTGGATATCACCTCCCACAACGAGGATTACACTATCGTTGAGCAATATGAAAGAGCTGAAGGGCGGCACTCGACAGGTGGCATGGATGAATTGTATAAG

mCherry 3 synthetic introns (ia, ib, ic)

ATGGTCTCAAAGGGTGAAGAAGATAACATGGCAATTATTAAAGAGTTTATGCGTTTCAAGGTGCATATGGAGGGATCTGTCAATGGGCATGAGTTTGAAATTGAAGGTGAAGGAGAAGGCCGACCATATGAGGGAACACAAACCGCAAAACTAAAGgtaagtttaaacatatatatactaactaaccctgattatttaaattttcagGTAACTAAAGGCGGACCATTACCATTCGCCTGGGACATCCTCTCTCCACAGTTCATGTATGGAAGTAAAGCTTATGTTAAACATCCGGCAGATATACCAGATTATTTGAAACTTTCATTCCCGGAGGGTTTTAAGTGGGAACGCGTAATGAATTTTGAAGACGGAGGAGTTGTTACAGTGACGCAAGACTCAAGgtaagtttaaacagttcggtactaactaaccatacatatttaaattttcagCCTCCAAGATGGAGAATTTATTTATAAAGTCAAACTTCGAGGAACGAATTTCCCCTCGGATGGACCTGTTATGCAGAAGAAGACTATGGGATGGGAAGCTTCAAGTGAAAGAATGTACCCTGAAGACGGTGCTCTTAAGGGAGAGATTAAACAACGTCTTAAATTGAAAGATGGAGGACATTACGATGCTGAGgtaagtttaaacatgattttactaactaactaatctgatttaaattttcagGTGAAGACAACTTACAAAGCCAAAAAACCAGTTCAGCTGCCAGGAGCGTACAATGTTAATATTAAACTGGATATCACCTCCCACAACGAGGATTACACTATCGTTGAGCAATATGAAAGAGCTGAAGGGCGGCACTCGACAGGTGGCATGGATGAATTGTATAAG

mCherry 2 hsp90 introns (id, ie)

ATGGTCTCAAAGGGTGAAGAAGATAACATGGCAATTATTAAAGAGTTTATGCGTTTCAAGGTGCATATGGAGGGATCTGTCAATGGGCATGAGTTTGAAATTGAAGGTGAAGGAGAAGGCCGACCATATGAGGGAACACAAACCGCAAAACTAAAGgtttgttttttcgcttctgagtcaattttttaaaatatcggttttagGTAACTAAAGGCGGACCATTACCATTCGCCTGGGACATCCTCTCTCCACAGTTCATGTATGGAAGTAAAGCTTATGTTAAACATCCGGCAGATATACCAGATTATTTGAAACTTTCATTCCCGGAGGGTTTTAAGTGGGAACGCGTAATGAATTTTGAAGACGGAGGAGTTGTTACAGTGACGCAAGACTCAAGgtatttttagttttaattattttatggcaataattccattaatttcagCCTCCAAGATGGAGAATTTATTTATAAAGTCAAACTTCGAGGAACGAATTTCCCCTCGGATGGACCTGTTATGCAGAAGAAGACTATGGGATGGGAAGCTTCAAGTGAAAGAATGTACCCTGAAGACGGTGCTCTTAAGGGAGAGATTAAACAACGTCTTAAATTGAAAGATGGAGGACATTACGATGCTGAGGTGAAGACAACTTACAAAGCCAAAAAACCAGTTCAGCTGCCAGGAGCGTACAATGTTAATATTAAACTGGATATCACCTCCCACAACGAGGATTACACTATCGTTGAGCAATATGAAAGAGCTGAAGGGCGGCACTCGACAGGTGGCATGGATGAATTGTATAAG

mCherry 2 synthetic introns (ia, ib)

ATGGTCTCAAAGGGTGAAGAAGATAACATGGCAATTATTAAAGAGTTTATGCGTTTCAAGGTGCATATGGAGGGATCTGTCAATGGGCATGAGTTTGAAATTGAAGGTGAAGGAGAAGGCCGACCATATGAGGGAACACAAACCGCAAAACTAAAGgtaagtttaaacatatatatactaactaaccctgattatttaaattttcagGTAACTAAAGGCGGACCATTACCATTCGCCTGGGACATCCTCTCTCCACAGTTCATGTATGGAAGTAAAGCTTATGTTAAACATCCGGCAGATATACCAGATTATTTGAAACTTTCATTCCCGGAGGGTTTTAAGTGGGAACGCGTAATGAATTTTGAAGACGGAGGAGTTGTTACAGTGACGCAAGACTCAAGgtaagtttaaacagttcggtactaactaaccatacatatttaaattttcagCCTCCAAGATGGAGAATTTATTTATAAAGTCAAACTTCGAGGAACGAATTTCCCCTCGGATGGACCTGTTATGCAGAAGAAGACTATGGGATGGGAAGCTTCAAGTGAAAGAATGTACCCTGAAGACGGTGCTCTTAAGGGAGAGATTAAACAACGTCTTAAATTGAAAGATGGAGGACATTACGATGCTGAGGTGAAGACAACTTACAAAGCCAAAAAACCAGTTCAGCTGCCAGGAGCGTACAATGTTAATATTAAACTGGATATCACCTCCCACAACGAGGATTACACTATCGTTGAGCAATATGAAAGAGCTGAAGGGCGGCACTCGACAGGTGGCATGGATGAATTGTATAAG

mCherry 1 synthetic intron 5’ end (ia)

ATGGTCTCAAAGGGTGAAGAAGATAACATGGCAATTATTAAAGAGTTTATGCGTTTCAAGGTGCATATGGAGGGATCTGTCAATGGGCATGAGTTTGAAATTGAAGGTGAAGGAGAAGGCCGACCATATGAGGGAACACAAACCGCAAAACTAAAGgtaagtttaaacatatatatactaactaaccctgattatttaaattttcagGTAACTAAAGGCGGACCATTACCATTCGCCTGGGACATCCTCTCTCCACAGTTCATGTATGGAAGTAAAGCTTATGTTAAACATCCGGCAGATATACCAGATTATTTGAAACTTTCATTCCCGGAGGGTTTTAAGTGGGAACGCGTAATGAATTTTGAAGACGGAGGAGTTGTTACAGTGACGCAAGACTCAAGCCTCCAAGATGGAGAATTTATTTATAAAGTCAAACTTCGAGGAACGAATTTCCCCTCGGATGGACCTGTTATGCAGAAGAAGACTATGGGATGGGAAGCTTCAAGTGAAAGAATGTACCCTGAAGACGGTGCTCTTAAGGGAGAGATTAAACAACGTCTTAAATTGAAAGATGGAGGACATTACGATGCTGAGGTGAAGACAACTTACAAAGCCAAAAAACCAGTTCAGCTGCCAGGAGCGTACAATGTTAATATTAAACTGGATATCACCTCCCACAACGAGGATTACACTATCGTTGAGCAATATGAAAGAGCTGAAGGGCGGCACTCGACAGGTGGCATGGATGAATTGTATAAG

mCherry 1 synthetic intron 3’ end (ia)

ATGGTCTCAAAGGGTGAAGAAGATAACATGGCAATTATTAAAGAGTTTATGCGTTTCAAGGTGCATATGGAGGGATCTGTCAATGGGCATGAGTTTGAAATTGAAGGTGAAGGAGAAGGCCGACCATATGAGGGAACACAAACCGCAAAACTAAAGGTAACTAAAGGCGGACCATTACCATTCGCCTGGGACATCCTCTCTCCACAGTTCATGTATGGAAGTAAAGCTTATGTTAAACATCCGGCAGATATACCAGATTATTTGAAACTTTCATTCCCGGAGGGTTTTAAGTGGGAACGCGTAATGAATTTTGAAGACGGAGGAGTTGTTACAGTGACGCAAGACTCAAGCCTCCAAGATGGAGAATTTATTTATAAAGTCAAACTTCGAGGAACGAATTTCCCCTCGGATGGACCTGTTATGCAGAAGAAGACTATGGGATGGGAAGCTTCAAGTGAAAGAATGTACCCTGAAGACGGTGCTCTTAAGGGAGAGATTAAACAACGTCTTAAATTGAAAGATGGAGGACATTACGATGCTGAGgtaagtttaaacatatatatactaactaaccctgattatttaaattttcagGTGAAGACAACTTACAAAGCCAAAAAACCAGTTCAGCTGCCAGGAGCGTACAATGTTAATATTAAACTGGATATCACCTCCCACAACGAGGATTACACTATCGTTGAGCAATATGAAAGAGCTGAAGGGCGGCACTCGACAGGTGGCATGGATGAATTGTATAAG

mCherry 1 synthetic intron 5’ end (ic)

ATGGTCTCAAAGGGTGAAGAAGATAACATGGCAATTATTAAAGAGTTTATGCGTTTCAAGGTGCATATGGAGGGATCTGTCAATGGGCATGAGTTTGAAATTGAAGGTGAAGGAGAAGGCCGACCATATGAGGGAACACAAACCGCAAAACTAAAGgtaagtttaaacatgattttactaactaactaatctgatttaaattttcagGTAACTAAAGGCGGACCATTACCATTCGCCTGGGACATCCTCTCTCCACAGTTCATGTATGGAAGTAAAGCTTATGTTAAACATCCGGCAGATATACCAGATTATTTGAAACTTTCATTCCCGGAGGGTTTTAAGTGGGAACGCGTAATGAATTTTGAAGACGGAGGAGTTGTTACAGTGACGCAAGACTCAAGCCTCCAAGATGGAGAATTTATTTATAAAGTCAAACTTCGAGGAACGAATTTCCCCTCGGATGGACCTGTTATGCAGAAGAAGACTATGGGATGGGAAGCTTCAAGTGAAAGAATGTACCCTGAAGACGGTGCTCTTAAGGGAGAGATTAAACAACGTCTTAAATTGAAAGATGGAGGACATTACGATGCTGAGGTGAAGACAACTTACAAAGCCAAAAAACCAGTTCAGCTGCCAGGAGCGTACAATGTTAATATTAAACTGGATATCACCTCCCACAACGAGGATTACACTATCGTTGAGCAATATGAAAGAGCTGAAGGGCGGCACTCGACAGGTGGCATGGATGAATTGTATAAG

mCherry 1 hsp90 intron 5’ end (id)

ATGGTCTCAAAGGGTGAAGAAGATAACATGGCAATTATTAAAGAGTTTATGCGTTTCAAGGTGCATATGGAGGGATCTGTCAATGGGCATGAGTTTGAAATTGAAGGTGAAGGAGAAGGCCGACCATATGAGGGAACACAAACCGCAAAACTAAAGgtttgttttttcgcttctgagtcaattttttaaaatatcggttttagGTAACTAAAGGCGGACCATTACCATTCGCCTGGGACATCCTCTCTCCACAGTTCATGTATGGAAGTAAAGCTTATGTTAAACATCCGGCAGATATACCAGATTATTTGAAACTTTCATTCCCGGAGGGTTTTAAGTGGGAACGCGTAATGAATTTTGAAGACGGAGGAGTTGTTACAGTGACGCAAGACTCAAGCCTCCAAGATGGAGAATTTATTTATAAAGTCAAACTTCGAGGAACGAATTTCCCCTCGGATGGACCTGTTATGCAGAAGAAGACTATGGGATGGGAAGCTTCAAGTGAAAGAATGTACCCTGAAGACGGTGCTCTTAAGGGAGAGATTAAACAACGTCTTAAATTGAAAGATGGAGGACATTACGATGCTGAGGTGAAGACAACTTACAAAGCCAAAAAACCAGTTCAGCTGCCAGGAGCGTACAATGTTAATATTAAACTGGATATCACCTCCCACAACGAGGATTACACTATCGTTGAGCAATATGAAAGAGCTGAAGGGCGGCACTCGACAGGTGGCATGGATGAATTGTATAAG

mEGP no introns

ATGAGTAAAGGAGAAGAACTTTTCACTGGAGTTGTCCCAATTCTTGTTGAATTAGATGGTGATGTTAATGGGCACAAATTTTCTGTCAGTGGAGAGGGTGAAGGTGATGCAACATACGGAAAACTTACCCTTAAATTTATTTGCACTACTGGAAAACTACCTGTTCCATGGCCAACACTTGTCACTACTTTCACTTATGGTGTTCAATGCTTTTCAAGATACCCAGATCATATGAAACgGCATGACTTTTTCAAGAGTGCCATGCCCGAAGGTTATGTACAGGAAAGAACTATATTTTTCAAAGATGACGGGAACTACAAGACACGTGCTGAAGTCAAGTTTGAAGGTGATACCCTTGTTAATAGAATCGAGTTAAAAGGTATTGATTTTAAAGAAGATGGAAACATTCTTGGACACAAATTGGAATACAACTATAACTCACACAATGTATACATCATGGCAGACAAACAAAAGAATGGAATCAAAGTTAACTTCAAAACTAGACACAACATTGAAGATGGAAGCGTTCAACTAGCAGACCATTATCAACAAAATACTCCAATTGGCGATGGCCCTGTCCTTTTACCAGACAACCATTACCTGTCCACACAATCTAAGCTTTCGAAAGATCCCAACGAAAAGAGAGACCACATGGTCCTTCTTGAGTTTGTAACAGCTGCTGGGATTACACATGGCATGGATGAACTATACAAA

mEGFP 3 synthetic introns (ia, ib, ic)

ATGAGTAAAGGAGAAGAACTTTTCACTGGAGTTGTCCCAATTCTTGTTGAATTAGATGGTGATGTTAATGGGCACAAATTTTCTGTCAGTGGAGAGGGTGAAGGTGATGCAACATACGGAAAACTTACCCTTAAATTTATTTGCACTACTGGAAAACTACCTGTTCCATGGgtaagtttaaacatatatatactaactaaccctgattatttaaattttcagCCAACACTTGTCACTACTTTCACTTATGGTGTTCAATGCTTTTCAAGATACCCAGATCATATGAAACgGCATGACTTTTTCAAGAGTGCCATGCCCGAAGGTTATGTACAGGAAAGAACTATATTTTTCAAAGATGACGGGAACTACAAGACACgtaagtttaaacagttcggtactaactaaccatacatatttaaattttcagGTGCTGAAGTCAAGTTTGAAGGTGATACCCTTGTTAATAGAATCGAGTTAAAAGGTATTGATTTTAAAGAAGATGGAAACATTCTTGGACACAAATTGGAATACAACTATAACTCACACAATGTATACATCATGGCAGACAAACAAAAGAATGGAATCAAAGTTgtaagtttaaacatgattttactaactaactaatctgatttaaattttcagAACTTCAAAACTAGACACAACATTGAAGATGGAAGCGTTCAACTAGCAGACCATTATCAACAAAATACTCCAATTGGCGATGGCCCTGTCCTTTTACCAGACAACCATTACCTGTCCACACAATCTAAGCTTTCGAAAGATCCCAACGAAAAGAGAGACCACATGGTCCTTCTTGAGTTTGTAACAGCTGCTGGGATTACACATGGCATGGATGAACTATACAAA

**Statistical Analyses**

Statistical Analyses Performed in Crane et al.

**One Way Analysis of Variance**

**Data source:** Phsp-90::mCherry Expression Levels with different intron configurations

**Normality Test (Shapiro-Wilk)** Failed (P < 0.050)

**Kruskal-Wallis One Way Analysis of Variance on Ranks**

**Data source:** Phsp-90::mCherry Expression Levels with different intron configurations

**Group N Missing Median 25% 75%**

2631 297 0 1064.700 1000.500 1124.100 <- Intronless Control

2721 179 0 1579.700 1497.700 1685.600

2642 185 0 1541.200 1419.600 1687.200

2751 165 0 1101.300 1038.300 1156.150

2711 210 0 1573.950 1420.100 1675.350

2731 135 0 1553.600 1448.900 1627.000

2741 102 0 1530.750 1467.225 1619.800

2761 195 0 1512.800 1436.600 1608.500

H = 879.129 with 7 degrees of freedom. (P = <0.001)

The differences in the median values among the treatment groups are greater than would be expected by chance; there is a statistically significant difference (P = <0.001)

To isolate the group or groups that differ from the others use a multiple comparison procedure.

Multiple Comparisons versus Control Group (Dunn's Method) :

**Comparison Diff of Ranks Q P<0.05**

2721 vs 2631 794.958 19.818 Yes

2642 vs 2631 727.193 18.315 Yes

2731 vs 2631 725.049 16.477 Yes

2741 vs 2631 710.626 14.607 Yes

2711 vs 2631 688.114 18.004 Yes

2761 vs 2631 666.493 17.058 Yes

2751 vs 2631 43.177 1.049 No

Note: The multiple comparisons on ranks do not include an adjustment for ties.

**One Way Analysis of Variance**

**Data source:** Phsp-90::mCherry Expression levels with different intron configurations

**Normality Test (Shapiro-Wilk)** Failed (P < 0.050)

**Kruskal-Wallis One Way Analysis of Variance on Ranks**

**Data source:** Phsp-90::mCherry Expression levels with different intron configurations

**Group N Missing Median 25% 75%**

2631 297 0 1064.700 1000.500 1124.100

2721 179 0 1579.700 1497.700 1685.600

2642 185 0 1541.200 1419.600 1687.200

2751 165 0 1101.300 1038.300 1156.150

2711 210 0 1573.950 1420.100 1675.350

2731 135 0 1553.600 1448.900 1627.000

2741 102 0 1530.750 1467.225 1619.800

2761 195 0 1512.800 1436.600 1608.500

H = 879.129 with 7 degrees of freedom. (P = <0.001)

The differences in the median values among the treatment groups are greater than would be expected by chance; there is a statistically significant difference (P = <0.001)

To isolate the group or groups that differ from the others use a multiple comparison procedure.

All Pairwise Multiple Comparison Procedures (Dunn's Method) :

**Comparison Diff of Ranks Q P<0.05**

2721 vs 2631 794.958 19.818 Yes

2721 vs 2751 751.781 16.432 Yes

2721 vs 2761 128.465 2.928 No

2721 vs 2711 106.844 2.478 Do Not Test

2721 vs 2741 84.332 1.604 Do Not Test

2721 vs 2731 69.908 1.447 Do Not Test

2721 vs 2642 67.765 1.525 Do Not Test

2642 vs 2631 727.193 18.315 Yes

2642 vs 2751 684.016 15.069 Yes

2642 vs 2761 60.700 1.395 Do Not Test

2642 vs 2711 39.079 0.914 Do Not Test

2642 vs 2741 16.567 0.317 Do Not Test

2642 vs 2731 2.143 0.0447 Do Not Test

2731 vs 2631 725.049 16.477 Yes

2731 vs 2751 681.873 13.860 Yes

2731 vs 2761 58.556 1.234 Do Not Test

2731 vs 2711 36.936 0.790 Do Not Test

2731 vs 2741 14.424 0.259 Do Not Test

2741 vs 2631 710.626 14.607 Yes

2741 vs 2751 667.449 12.500 Yes

2741 vs 2761 44.133 0.852 Do Not Test

2741 vs 2711 22.512 0.440 Do Not Test

2711 vs 2631 688.114 18.004 Yes

2711 vs 2751 644.937 14.624 Yes

2711 vs 2761 21.621 0.513 Do Not Test

2761 vs 2631 666.493 17.058 Yes

2761 vs 2751 623.316 13.901 Yes

2751 vs 2631 43.177 1.049 No

**One Way Analysis of Variance**

**Data source:** Phsp-90::mEGFP Expression levels with and without introns

Dependent Variable: Expression level

**Normality Test (Shapiro-Wilk)** Failed (P < 0.050)

**Kruskal-Wallis One Way Analysis of Variance on Ranks**

**Data source: Phsp-90::mEGFP Expression levels with and without introns**

**Group N Missing Median 25% 75%**

no introns 215 0 2222.100 2015.300 2405.700

introns 260 0 4078.250 3743.325 4357.400

H = 349.543 with 1 degrees of freedom. (P = <0.001)

The differences in the median values among the treatment groups are greater than would be expected by chance; there is a statistically significant difference (P = <0.001)

To isolate the group or groups that differ from the others use a multiple comparison procedure.

All Pairwise Multiple Comparison Procedures (Dunn's Method) :

**Comparison Diff of Ranks Q P<0.05**

introns vs no introns 236.565 18.696 Yes

Note: The multiple comparisons on ranks do not include an adjustment for ties.

**Two Way Analysis of Variance**

Phsp-90::mEGFP

General Linear Model

Dependent Variable: Expression level

**Normality Test (Shapiro-Wilk)** Failed (P < 0.050)

**Equal Variance Test:** Failed (P < 0.050)

**Source of Variation DF SS MS F P**

Experiment 2 13419511.389 6709755.695 59.105 <0.001

Introns Present 1 391095587.762 391095587.762 3445.083 <0.001

Experiment x Introns Present 2 4846224.923 2423112.461 21.345 <0.001

Residual 469 53242202.277 113522.819

Total 474 468601805.358 988611.404

Main effects cannot be properly interpreted if significant interaction is determined. This is because the size of a factor's effect depends upon the level of the other factor.

The effect of different levels of Experiment depends on what level of Introns Present is present. There is a statistically significant interaction between Experiment and Introns Present. (P = <0.001)

Power of performed test with alpha = 0.0500: for Experiment : 1.000

Power of performed test with alpha = 0.0500: for Introns Present : 1.000

Power of performed test with alpha = 0.0500: for Experiment x Introns Present : 1.000

Least square means for Experiment :

**Group Mean SEM**

trial 1 3330.851 31.302

trial 2 3278.911 31.449

trial 3 2966.376 22.118

Least square means for Introns Present :

**Group Mean SEM**

no introns 2222.040 23.521

introns 4162.052 23.222

Least square means for Experiment x Introns Present :

**Group Mean SEM**

trial 1 x no introns 2231.345 43.498

trial 1 x introns 4430.357 45.024

trial 2 x no introns 2319.037 43.498

trial 2 x introns 4238.785 45.432

trial 3 x no introns 2115.739 34.568

trial 3 x introns 3817.012 27.603

All Pairwise Multiple Comparison Procedures (Holm-Sidak method):

Overall significance level = 0.05

Comparisons for factor: **Experiment**

**Comparison Diff of Means t P P<0.050**

trial 1 vs. trial 3 364.476 9.509 <0.001 Yes

trial 2 vs. trial 3 312.536 8.129 <0.001 Yes

trial 1 vs. trial 2 51.940 1.171 0.242 No

Comparisons for factor: **Introns Present**

**Comparison Diff of Means t P P<0.050**

introns vs. no introns 1940.011 58.695 <0.001 Yes

Comparisons for factor: **Introns Present within trial 1**

**Comparison Diff of Means t P P<0.05**

introns vs. no introns 2199.012 35.126 <0.001 Yes

Comparisons for factor: **Introns Present within trial 2**

**Comparison Diff of Means t P P<0.05**

introns vs. no introns 1919.749 30.522 <0.001 Yes

Comparisons for factor: **Introns Present within trial 3**

**Comparison Diff of Means t P P<0.05**

introns vs. no introns 1701.273 38.459 <0.001 Yes

Comparisons for factor: **Experiment within no introns**

**Comparison Diff of Means t P P<0.05**

trial 2 vs. trial 3 203.298 3.659 <0.001 Yes

trial 1 vs. trial 3 115.606 2.081 0.075 No

trial 2 vs. trial 1 87.692 1.426 0.155 No

Comparisons for factor: **Experiment within introns**

**Comparison Diff of Means t P P<0.05**

trial 1 vs. trial 3 613.345 11.614 <0.001 Yes

trial 2 vs. trial 3 421.773 7.934 <0.001 Yes

trial 1 vs. trial 2 191.572 2.995 0.003 Yes

**One Way Analysis of Variance**

**Data source:** Pvit-2::mCherry Expression Levels with and without introns

Dependent Variable: Expression level

**Normality Test (Shapiro-Wilk)** Failed (P < 0.050)

Test execution ended by user request, ANOVA on Ranks begun

**Kruskal-Wallis One Way Analysis of Variance on Ranks**

**Data source:** Pvit-2::mCherry Expression Levels with and without introns

**Group N Missing Median 25% 75%**

No Intnrons 134 0 2617.250 2015.375 2967.150

Introns 163 0 4067.000 3697.400 4408.100

H = 173.324 with 1 degrees of freedom. (P = <0.001)

The differences in the median values among the treatment groups are greater than would be expected by chance; there is a statistically significant difference (P = <0.001)

To isolate the group or groups that differ from the others use a multiple comparison procedure.

All Pairwise Multiple Comparison Procedures (Dunn's Method) :

**Comparison Diff of Ranks Q P<0.05**

Introns vs No Intnrons 131.843 13.165 Yes

Note: The multiple comparisons on ranks do not include an adjustment for ties.

**Two Way Analysis of Variance**

**Data source:** Pvit-2::mCherry Expression Levels with and without introns

General Linear Model

Dependent Variable: Col 1

**Normality Test (Shapiro-Wilk)** Failed (P < 0.050)

**Equal Variance Test:** Failed (P < 0.050)

**Source of Variation DF SS MS F P**

Experiment 2 33864654.911 16932327.456 53.437 <0.001

Introns Present 1 172080597.603 172080597.603 543.076 <0.001

Experiment x Introns Present 2 2995637.104 1497818.552 4.727 0.010

Residual 291 92207153.406 316863.070

Total 296 291590000.289 985101.352

Main effects cannot be properly interpreted if significant interaction is determined. This is because the size of a factor's effect depends upon the level of the other factor.

The effect of different levels of Experiment depends on what level of Introns Present is present. There is a statistically significant interaction between Experiment and Introns Present. (P = 0.010)

Power of performed test with alpha = 0.0500: for Experiment : 1.000

Power of performed test with alpha = 0.0500: for Introns Present : 1.000

Power of performed test with alpha = 0.0500: for Experiment x Introns Present : 0.680

Least square means for Experiment :

**Group Mean SEM**

experiment 1 3190.948 58.178

experiment 2 3815.335 61.899

Experiment 3 2981.843 54.503

Least square means for Introns Present :

**Group Mean SEM**

No Intnrons 2545.354 48.831

Introns 4113.397 46.292

Least square means for Experiment x Introns Present :

**Group Mean SEM**

experiment 1 x No Intnrons 2266.032 79.607

experiment 1 x Introns 4115.864 84.861

experiment 2 x No Intnrons 3144.386 84.861

experiment 2 x Introns 4486.285 90.137

Experiment 3 x No Intnrons 2225.645 89.003

Experiment 3 x Introns 3738.041 62.935

All Pairwise Multiple Comparison Procedures (Holm-Sidak method):

Overall significance level = 0.05

Comparisons for factor: **Experiment**

**Comparison Diff of Means t P P<0.050**

experiment 2 vs. Experiment 3 833.492 10.106 <0.001 Yes

experiment 2 vs. experiment 1 624.388 7.350 <0.001 Yes

experiment 1 vs. Experiment 3 209.105 2.623 0.009 Yes

Comparisons for factor: **Introns Present**

**Comparison Diff of Means t P P<0.050**

Introns vs. No Intnrons 1568.042 23.304 <0.001 Yes

Comparisons for factor: **Introns Present within experiment 1**

**Comparison Diff of Means t P P<0.05**

Introns vs. No Intnrons 1849.832 15.898 <0.001 Yes

Comparisons for factor: **Introns Present within experiment 2**

**Comparison Diff of Means t P P<0.05**

Introns vs. No Intnrons 1341.898 10.839 <0.001 Yes

Comparisons for factor: **Introns Present within Experiment 3**

**Comparison Diff of Means t P P<0.05**

Introns vs. No Intnrons 1512.396 13.874 <0.001 Yes

Comparisons for factor: **Experiment within No Intnrons**

**Comparison Diff of Means t P P<0.05**

experiment 2 vs. experiment 1 878.354 7.549 <0.001 Yes

experiment 2 vs. Experiment 3 918.741 7.471 <0.001 Yes

experiment 1 vs. Experiment 3 40.387 0.338 0.735 No

Comparisons for factor: **Experiment within Introns**

**Comparison Diff of Means t P P<0.05**

experiment 2 vs. Experiment 3 748.243 6.806 <0.001 Yes

experiment 1 vs. Experiment 3 377.822 3.576 <0.001 Yes

experiment 2 vs. experiment 1 370.421 2.992 0.003 Yes

**One Way Analysis of Variance**

**Data source:** IME effect sizes for different promoters and intron configurations

**Normality Test (Shapiro-Wilk)** Failed (P < 0.050)

**Kruskal-Wallis One Way Analysis of Variance on Ranks**

**Group N Missing Median 25% 75%**

Hsp-90 IME 185 0 1.438 1.324 1.574

Vit-2 IME 163 0 1.525 1.370 1.677

Hsp-90 ntrl IME 179 0 1.474 1.397 1.572

H = 13.073 with 2 degrees of freedom. (P = 0.001)

The differences in the median values among the treatment groups are greater than would be expected by chance; there is a statistically significant difference (P = 0.001)

To isolate the group or groups that differ from the others use a multiple comparison procedure.

All Pairwise Multiple Comparison Procedures (Dunn's Method) :

**Comparison Diff of Ranks Q P<0.05**

vit-2 intron vs daf-21 intron 59.100 3.613 Yes

vit-2 intron vs daf21 natural 33.457 2.029 No

daf21 natural vs daf-21 intron 25.643 1.606 No

Note: The multiple comparisons on ranks do not include an adjustment for ties.
